# A method for estimating wage, using standardised occupational classifications, for use in medical research in the place of self-reported income

**DOI:** 10.1186/1471-2288-14-59

**Published:** 2014-04-28

**Authors:** Tom Clemens, Chris Dibben

**Affiliations:** 1 School of Geography & Geosciences, University of St Andrews, St Andrews, UK; 2 School of Geosciences, University of Edinburgh, Edinburgh, UK

**Keywords:** Income, Synthetic data, Standard occupational classification, General health, Social survey

## Abstract

**Background:**

Income is predictive of many health outcomes and is therefore an important potential confounder to control for in studies. However it is often missing or poorly measured in epidemiological studies because of its complexity and sensitivity. This paper presents and validates an alternative approach to the survey collection of reported income through the estimation of a synthetic wage measure based on occupation.

**Methods:**

A synthetic measure of weekly wage was calculated using a multilevel random effects model of wage predicted by a Standard Occupational Classification (SOC) fitted in data from the UK Labour Force Survey (years 2001–2010)^a^. The estimates were validated and tested by comparing them to reported income and then contrasting estimated and reported income’s association with measures of health in the Scottish Health Survey (SHS) 2003 and wave one (2009) of the UK Household Longitudinal Study (UKHLS).

**Results:**

The synthetic estimates provided independent and additional explanatory power within models containing other traditional proxies for socio-economic position such as social class and small area based measures of socio-economic position. The estimates behaved very similarly to ‘real’, reported measures of both household and individual income when modelling a measure of ‘general health’.

**Conclusions:**

The findings suggest that occupation based synthetic estimates of wage are as effective in capturing the underlying relationship between income and health as survey reported income. The paper argues that the direct survey measurement of income in every study may not actually be necessary or indeed optimal.

## Background

The association between socio-economic position (SEP) and health is well established and adjusting for the confounding effect of SEP in epidemiological and medical research is common practice. Common measures of SEP include educational attainment, housing (tenure, conditions or amenities), a number of occupational based measures and income [1,2]. Of these measures, income is perhaps the best indicator of an individual’s material and wealth circumstances and has been linked to many health outcomes including mental health [3-6], mortality [7-10] and self-assessed health [11,12].

Income is a sensitive topic and a potentially complex question to answer for many individuals and this can result in measurement error or missing data in surveys. Non-response rates of around 10-25% are common [13,14]. Furthermore, the format of the question or the subject matter of the survey is also likely to affect accuracy [15]. By varying degrees, all of these factors are, therefore, likely to introduce bias [16] and whilst some of these difficulties can be overcome with the implementation of more sophisticated survey designs [17], in many cases these are expensive and difficult to implement [18]. In some cases, concerns over the impact of asking an income question on overall response rates means that an income question is not asked at all. For example, despite strong pressure from the research community [18-20] the UK census will continue to omit an income question because of the difficulties respondents faced answering the question, the effect on response rates and potential negative coverage in the national press [21].

Despite this, there has been relatively little use of detailed descriptions of occupation to approximate a measure of material disadvantage, beyond collapsing them into traditional social class measures, despite their ubiquity in many data sources. This is possibly because of the difficulties associated with meaningfully incorporating large numbers of different occupational categories into a statistical analysis. However, aggregating occupation groups into social classes will result in a significant loss of occupation related discrimination in terms of socio-economic position. This paper argues that the utilisation of detailed occupation information, and its conversion onto an estimated continuous monetary scale, offers the potential for improved adjustment for SEP in medical research over traditional proxy measures. The paper sets out an approach based on common and widely available occupation classification schemes such as the UK Standard Occupation Classification (SOC). In addition to being highly discriminating in terms of wages, the SOC and other similar measures such as the International Standard Classification of Occupations (ISCO) are relatively common in most social survey datasets and so the approach described here is generalizable to a wide range of datasets from different countries.

## Methods

### Modelling approach

**Figure 1:**
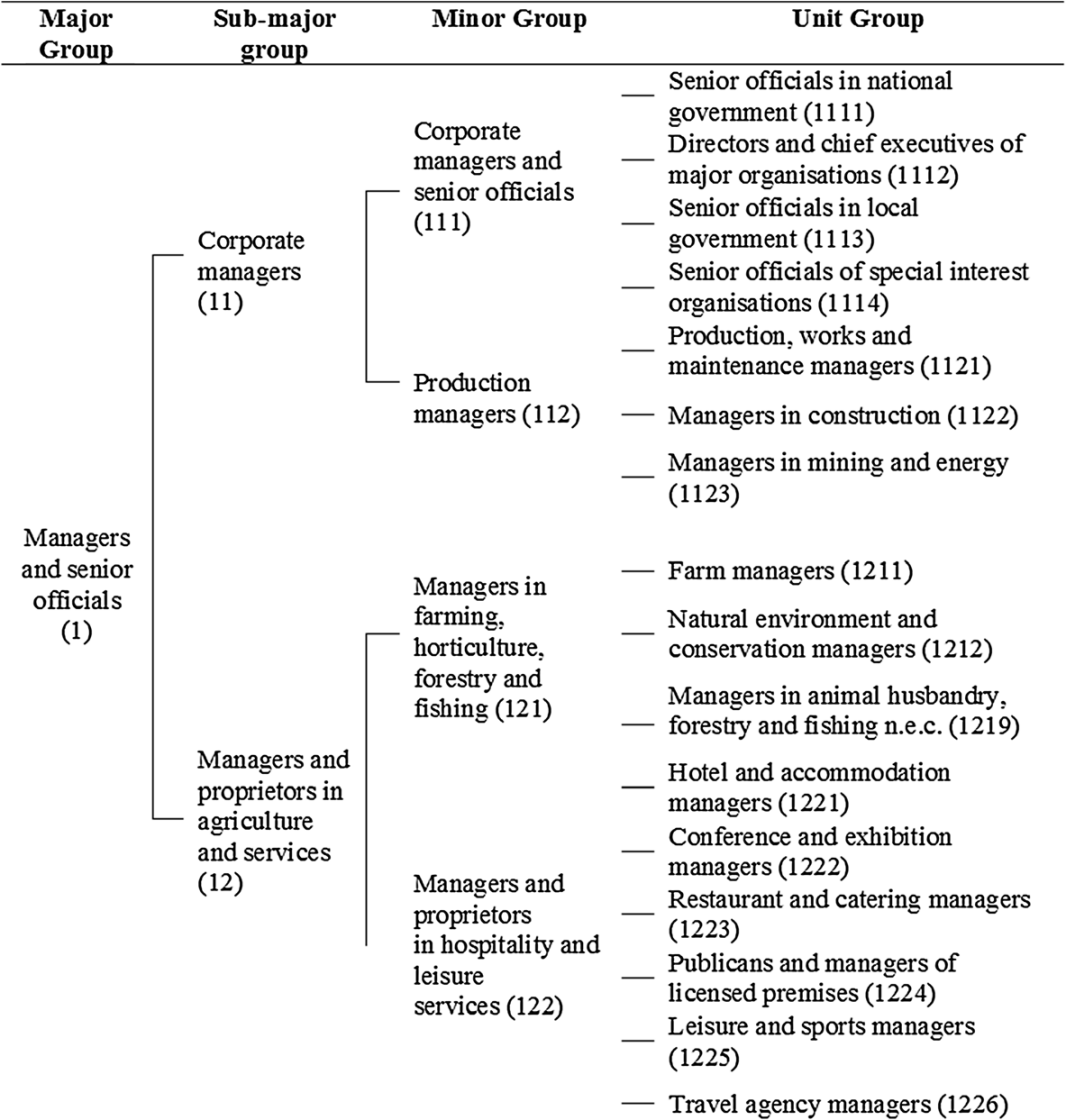
**Tiered structure of the standard occupation classification 2000 for select occupations (Managers and senior officials).** Diagram illustrates the different occupational groupings and levels that form the basis of the multilevel-models used to estimate synthetic wages. Numbers in brackets correspond to the occupation code. Diagram represents three of the eleven minor groupings within the managers and senior officials’ major grouping only due to space constraints. Details of the full classification can be found in ONS (2000).

We aimed to estimate wage among workers using the UK’s Standard Occupational Classification 2000 (SOC 2000) [22] which is a tiered classification of occupations developed by the UK’s Office of National Statistics (ONS). Figure 1 illustrates this structure for a sub-set of unit level occupations that fall under the major group of managers and senior officials. Each descending level provides increasingly detailed descriptions of occupational type, aimed at capturing the kind of work performed and the competence required to complete the tasks and duties associated with that work. Because wages tend to vary with these characteristics, SOC code would appear to be a potentially useful estimator of wage.

We used a multi-level mixed effects approach to model and then synthesise wages for working individuals by SOC units (the finest detailed grouping within the SOC). In order to ensure that the resulting estimates could be replicated in as wide a range of data sources as possible, it was decided to restrict the variables used in the models to age, sex and SOC occupation. The mixed effect models utilise the tiered structure of the SOC and estimate random effect parameters associated with each of the groups (levels) within corresponding tiers of the SOC together with fixed effect parameters for age and sex. Modelling was carried out in STATA version 11 and used maximum likelihood estimation within the xtmixed command. The ‘predicted’ wage (the synthetic estimate) was calculated in the survey data using the parameters from the original model including the fixed effects coefficients for age and sex (applied to age and sex information in the survey) together with the ‘shrunk’ multi-level residuals from each SOC group (corresponding to the SOC information in the survey) (see Additional file 1). The hierarchical nature of the SOC classification and the use of Empirical Bayes ‘shrunk’ multi-level residuals meant that the estimates were statistically efficient even where the sample size in any given SOC unit group was small.

### Data

#### Master data

Information on individual wage, from which the prediction models were estimated, was obtained from the UK Labour Force Survey (LFS). The LFS is a survey of approximately 50,000 households living at private addresses. It is collected every 3 months and is designed to be nationally representative of the UK population including all people resident in private households, all persons resident in National Health Service accommodation and young people living away from the parental home in a student hall of residence or similar institution during term time. We used data collected for the years 2001–2005 and 2007–2010. Data from 2006 was omitted to allow subsequent internal validation of the models. The wage information was derived from self-reported responses to a question asking for ‘gross weekly income in main job’ and was standardised to 2006 earnings levels using the consumer price index (CPI). The analysis sample was restricted to individuals of working age (16–65 for men and 16–60 for women) as pension type earnings could not be estimated reliably with occupation and to those individuals who were in employment in the week previous to the survey as those out of work were not assigned a SOC code. Outlying values in the wage distribution were determined by examining the skewness and were omitted together with those individuals who were missing information for wage, age, sex and SOC. These adjustments left a remaining sample size 251,537. For the modelling, wage values were log transformed in order to reduce the overall skewness in the distribution.

#### Validating data

Data from both the Scottish Health Survey (SHS) 2003 and wave one (2009) of the UK Household Longitudinal Study (UKHLS) were used to test the external validity of the synthetic estimate. The SHS has a total sample size of 11,472 and the UKHLS 22,265 and, similarly to the LFS, both surveys are designed to be representative of the population and cover similar age ranges. The surveys were chosen because they both contain information on self-rated general health as well as a large amount of demographic information including SOC, social class and income. The SHS has a measure of equivalised household income and the UKHLS has individual ‘take home’ wages. This then allowed a comparison between both of these measures and our synthetic estimate of wage. Samples were restricted to individuals with complete information for the variables in the analysis and to those aged 16 or over.

### Validation

#### Internal validation

The most effective model configuration was determined by examining the wage predictions generated in the 2006 labour force survey data which were evaluated by calculating the standard deviation of the residuals of the predicted wage subtracted from the actual wage.

#### External validation of the wage estimates

A comparison amongst working individuals between our estimate of wage against reported individual salary and household income when predicting health status was carried out using the SHS and UKHLS. This comparison was made using both grouped and continuous versions of the wage and income variables. In each case we examined the strength of the relationship between income or wage and general health. The general health variable in the SHS was assessed with the question “how is your health in general?” with responses very good, good, fair, bad or very bad and in the UKHLS the question was “In general, would you say your health is?” with responses excellent, very good, good, fair and poor. These variables were coded into binary indicators with the SHS comparing those with fair, bad or very bad health with those with good or very good health and the UKHLS data comparing those with fair or poor health with those with good or better health. Because the outcomes in both cases were binary, logistic regression was used to estimate model parameters and a method proposed by Zheng and Agresti [23] using correlations was used to compare model fit (the predicted values for the outcome are correlated with the observed values - the higher the correlation the better the fit of the model).

## Results

### Estimation of the prediction equations

Table 1 shows the results of the mixed models that were fitted to the master data from the LFS. Across all models the fixed effects for age and sex and the grand constant were all significant predictors of wages and the magnitude of the effects was mostly consistent, with wage increasing with age and being male. In model one there was significant variation in the random intercepts of each of the SOC minor groups around the fixed grand constant indicating that wages differed across SOC minor groups. Allowing the slopes of the SOC minor categories to vary with age in model two did not substantially reduce the amount of unexplained variance. In model three the overall fit of the model was improved with the addition of level 2 variance. In the final model, the level 2 & 3 intercepts retained significant variability though the variation in the age slopes of the lines appeared strongest at level 2.

**Table 1:** Details of different prediction models fitted to the master data predicting log weekly wage fixed and random effect parameters (to two significant figures) are reported together with overall residual variance of the model

**Parameter**	**Model 1: 2-level random intercepts (individuals nested in SOC minor groups)**	**Model 2: 2-level random intercepts and age slopes (individuals nested in SOC minor groups)**	**Model 3: 3-level random intercepts (individuals nested in SOC unit groups nested in SOC minor groups)**	**Model 4: 3-level random intercepts and age slopes (individuals nested in SOC unit groups nested in SOC minor groups)**
**Fixed effects**^**#**^				
Age (increments of one year)	0.0064	0.0052	0.0063	0.005
Sex (female reference)	0.31	0.33	0.26	0.27
Intercept	5.00034	5.04	5.1	5.14
**Random effects**^**$**^				
Level - SOC minor				
Intercept	0.16	0.26	0.13	0.14
Slope (age)		0.00003		0.0
Level - SOC unit				
Intercept			0.05	0.1
Slope (age)				0.00003
**Residual (variance)**	0.31	0.31	0.29	0.28
**N (for all models)**	251,537			

Fixed and random effect parameters (to two significant figures) are reported together with overall residual variance of the model.

^#^Fixed effect parameters are reported on the log wage scale.

^$^Random effects parameters are reported on the log wage scale and show the standard deviation of the estimated intercepts and slope coefficients at each level.

### Internal validation of the prediction equations

The mean for the actual weekly wage in the 2006 LFS data was £356. Table 2 displays the deviations of the wage predictions from their actual values reported in the 2006 LFS. Model 4 has the closest fit to the actual wage data. Comparing the intercept only models (Models one and three), adding a random intercept for level three (the SOC minor grouping) improved the accuracy by an average of around £5 (calculated by subtracting the deviation in model 1 from model 3). Improvement with the addition of varying age slopes was less marked. Model 4 improved the accuracy of the predictions by around £65 (deviation from model 4 subtracted from deviation when using simple geometric mean) per person compared to the single valued geometric mean. Model 4 was therefore used to construct the wage estimate.

**Table 2:** Evaluation (using average deviation of the predicted wage from actual wage and% reduction in total deviation) of both prediction models and simple geometric means (grand mean and mean within SOC unit categories) for the internal validation data (2006 LFS data only)

**Model**	**Average deviation from actual wage**	**% reduction of deviation**
**Grand Geometric Mean Wage**	£209	0%
**Geometric Mean wage in SOC Unit Group**	£150	48.78%
**Model 1: 2-level intercept**	£151	48.12%
**Model 2: 2-level intercept and age slopes**	£150	48.41%
**Model 3: 3-level intercept**	£146	51.63%
**Model 4: 3-level intercept and age slopes**	£145	52.10%
**N**	27,560	

### External validation

#### Comparison of synthetic estimates to other measures of SEP

The synthetic wage estimates were calculated in the SHS using model 4. Table 3 presents results showing the effect of adjusting for SEP for both the synthetic estimates and reported income (equivalised household income). In all four models, both measures significantly reduce the risk of reporting fair, poor or very poor general health. Although the synthetic wage effect attenuates in models two to five with the addition of social class (NSSEC and the registrar generals classification) and small area deprivation (as measured by the Scottish Index of Multiple Deprivation for 2006), a significant residual effect remains. The synthetic measure of wage was, even in model 4, a stronger predictor of self-reported health than the stated income measure.

**Table 3:** Comparison of synthetic wage and measured equivalised household income coefficients from models predicting fair, bad or very bad health estimated from Scottish Health Survey, adjusting for other measures of socio-economic position

	**N**	**Wage (synthetic estimate)**	**N**	**Reported equivalised income**		
		**Odds ratio of poor health**	**95% CI**		**Odds ratio of poor health**	**95% CI**
1. Wage (scaled in units of £100)-controlling for Age and Sex	7757	0.661***	0.623,0.702	7075	0.829***	0.810, 0.848
2. As 1 with additional control for social class	7749	0.790***	0.726,0.861	6865	0.866***	0.846, 0.888
3. As 2 with additional control for SIMD	7749	0.819***	0.751,0.893	6865	0.894***	0.872, 0.916
4. As 1 with additional control for SIMD	7757	0.735***	0.691,0.783	7075	0.870***	0.850, 0.891
5. As 2 with additional control for NSSEC8 and SIMD	7749	0.857**	0.779,0.943	6865	0.898***	0.877, 0.920

P-value *(p < .10) **(p < .05) *** (p < .01).

SIMD – Scottish Index of Multiple Deprivation: http://www.scotland.gov.uk/Topics/Statistics/SIMD.

NSSEC8 – 8 fold UK National Statistics Socio-economic Classification: http://www.ons.gov.uk/ons/guide-method/classifications/current-standard-classifications/soc2010/soc2010-volume-3-ns-sec--rebased-on-soc2010--user-manual/index.html.

#### Comparison of synthetic estimates with household and individual income

Table 4 presents the results of age and sex adjusted logistic regression models predicting fair or poor general health in order to compare the synthetic estimates to both individual and household survey measured income from the SHS and the UKHLS respectively. We also examine these income and wage measures in both continuous and discrete deciled form. Both of the continuous measures have a significant effect on self-reported health, with increases in each associated with a decline in the reporting of poor health. This effect is stronger for the synthetic wage measures. Similarly, for the grouped variables there is a clear gradient between the lowest and highest earning groups, with the highest earners significantly less likely to report poor health than those in the lowest earning group for both the synthetic estimates and the survey measured income. The magnitude of the coefficients differs slightly between models, particularly at lower income levels where they are insignificant for self-reported income but not for the synthetic measure. The correlation values show that, despite a weaker effect, survey measured continuous household income has a slightly better fit when compared to the synthetic model. The corresponding figures for the discrete analysis show similar patterns.

**Table 4:** **Model coefficients for synthetic wage and survey reported income (continuous and deciled) for age and sex adjusted logistic regression models predicting fair, bad or very bad health in Scottish Health Survey**[1]**reported income is equivalised household income and in UK household Longitudinal Study**[2]**individual wage**

	**Scottish Health Survey**[1]				**UK Household Longitudinal Study**[2]			
	**N**	**Odds ratio for poor health**	**95% CI**	**Corr (r)**	**N**	**Odds ratio for poor health**	**95% CI**	**Corr (r)**
Continuous Income (scaled in units of £100)								
Synthetic wage	7757	0.661***	0.623,0.702	0.288***	12457	0.765***	0.721,0.812	0.118***
Survey reported income	7075	0.829***	0.810,0.848	0.335***	9459	0.936***	0.910,0.962	0.098***
Deciled Income								
Synthetic wage (lowest income decile as reference)	7757			0.288***	12457			0.123***
1st		1				1		
2^nd^		0.678***	0.541,0.851			0.869	0.690,1.095	
3^rd^		0.725***	0.579,0.907			0.904	0.720,1.135	
4^th^		0.590***	0.468,0.743			0.759*	0.601,0.960	
5^th^		0.572***	0.455,0.720			0.742*	0.585,0.940	
6^th^		0.491***	0.386,0.623			0.539***	0.419,0.694	
7^th^		0.390***	0.305,0.499			0.542***	0.422,0.695	
8^th^		0.371***	0.290,0.475			0.490***	0.379,0.633	
9^th^		0.284***	0.220,0.366			0.549***	0.427,0.706	
Most		0.204***	0.155,0.268			0.377***	0.285,0.498	
Survey reported income (lowest income decile as reference)	7075			0.350***	9459			0.134***
1st		1				1		
2^nd^		1.204	0.947,1.529			0.844	0.656,1.086	
3^rd^		0.899	0.716,1.128			0.640***	0.492,0.833	
4^th^		0.714***	0.563,0.905			0.870	0.678,1.118	
5^th^		0.634***	0.498,0.807			0.695**	0.536,0.903	
6^th^		0.449***	0.347,0.582			0.594***	0.456,0.773	
7^th^		0.335***	0.260,0.431			0.437***	0.330,0.578	
8^th^		0.227***	0.171,0.301			0.320***	0.235,0.437	
9^th^		0.222***	0.167,0.296			0.497***	0.377,0.656	
Most		0.168***	0.124,0.226			0.419***	0.315,0.557	

Correlation measures the association between the predicted risk of poor health and the actual data comparable measure of model fit.

P-value *(p < .10) **(p < .05) *** (p < .01).

When comparing the synthetic estimates with an individual survey income measure the relative patterns between models differ from those examining household income. Firstly, the correlation values suggest much smaller differences in the fit of both the discrete and continuous models between the synthetic wage and real income variables. In the continuous models, the fit is actually marginally better when using the synthetic measure and, similarly, in comparison with the household income, has a stronger effect on general health. In terms of the discrete variable, the gradient pattern was less marked particularly for the real income model which also showed perhaps a slightly shallower gradient when compared to the synthetic model. It is worth noting that the two surveys asked the general health question in different ways and this may explain the difference between surveys in the various proportions of the population stating they experience good or poor health.

## Discussion

The collection of income information on a questionnaire or within time limited interview situations is not straightforward. This is reflected in both its absence from some research instruments, its simplified form in others and more generally its relatively high level of missing or improbable responses. In studies where a measure of income is entirely missing, the use of other indicators of socio-economic position such as social class, educational attainment or small area based indicators are frequently used to approximate the material disadvantage that would have been captured by an income measure. This study has proposed and examined an alternative approach, the estimation of a synthetic measure of individual wages among workers based on detailed occupation groups from a standard occupation classification. While occupation forms a key component of many social class based measures, this often involves collapsing detailed occupational categories to such an extent that much ‘information’ is lost. We utilised this ‘information’ to estimate a synthetic measure of occupational based wage and then tested its external validity in relation to the prediction of an often used self-reported general health measure. We observed two main findings. Firstly, the estimates provide independent and additional explanatory power within models containing only social class and small area based measures of socio-economic position alone and secondly, that they behaved very similarly to ‘real’, reported measures of both household and individual income when modelling ‘general health’. These findings suggest that occupation may be a useful variable with which to estimate a synthetic measure of wages and may provide a reliable and effective alternative or supplement for the recording of reported income in social and health surveys.

The approach we have taken has a number of advantages both when datasets are missing an income measure entirely as well as for those where it is imprecisely measured. In the former case, our findings appear to support the notion that wage measures a different component of SEP than that captured by social class and small area poverty or deprivation measures. This suggests that social class and small area deprivation on their own may not be sufficient to adjust for all socio-economic differences in general health and certainly not those differences that are related to income.

It could be argued that the synthetic occupation-based wage estimates also provide a more analytically useful measure of ‘average income’. Research suggests that of the many aspects of SEP, income is perhaps the component with the greatest degree of short-term variability [7] which means that a traditional cross sectional survey, collecting the data at a single time point, may not capture the underlying information of interest. Because our estimates are closer to an individual’s medium term average wage given their occupation, it may capture important economic forces more effectively than reported measures of income for a specific period of time. This may explain why our synthetic measure has better discrimination (in terms of health) at lower levels than reported income (see Table 4 – odds ratio for deciles). At this point in the income distribution casual employment with more variable rates of wage within any period of time will be more common and therefore a single sample in time may provide a poor estimate of average wage, the more important factor in the determination of health.

The methodology can be applied to a wide range of studies or datasets because the estimation models are reasonably parsimonious and only require a record of age, sex and occupation coded within some form of hierarchical or tiered standard classification. In most datasets these variables are unlikely to contain significant numbers of missing cases leading to mostly ignorable and negligible missing cases in the resulting estimates. It may also be possible to simplify the model further. Provided a sufficiently large dataset is available (ie sufficient number of cases in each occupation group), the mean wage level within an occupational group may provide as good an estimate of wage as the empirical bayes estimate used in this study (see Table 2).

The findings have a number of important implications for understanding confounding by socio-economic position and the collection of income data in surveys for health research. Firstly, it is clear that other non-income measures or components of SEP do not entirely capture the effect of income on their own and that omitting an income measure risks introducing income-related confounding. This is particularly problematic in datasets in which income is not measured such as the UK census and census based longitudinal studies. Extending this argument further, the findings may have wider implications for the measurement of income in health surveys more generally. Although we have restricted our analysis to an examination of self-reported health, the evidence begins to suggest that the collection of reported income data in health surveys may not be as crucial as the measurement of occupation. This is important as occupation is a far easier characteristic to measure and is much less problematic in terms of missing data, mis-measurement or inaccuracy.

There are limitations with the approach that we have used. Firstly, it relies on occupational information being available for subjects and, if household income needs to be calculated, for all those contributing to the household budget. Secondly, for those of working age, who are not employed or those who have retired, a description of occupation, if available, will not necessarily be an accurate measure of their income. However, it is possible to estimate the likely income for those who are unemployed or retired by using the standard welfare payments or occupational related pension payments. For those who have retired but have a pre-retirement occupation recorded, a similar modelling approach could be used to estimate pension level. Finally, the study was restricted to an examination of a measure of self-reported general health and it does not necessarily follow that our findings can be generalised to other health variables. For example, the shape, magnitude and functional form of the relationship between income and other health indicators such as mortality and physical health measures differs markedly in some cases [24-26]. It is important for future research to examine the validity of these synthetic estimates in relation to other health variables.

## Conclusion

This study suggests that a synthetic measure of wage based on occupation can be used as an effective alternative to self-reported income in health studies. Given the problems associated with questions about income in a survey context, for example its non- or inaccurate completion and its negative effect on overall response rates, this study also suggests that it may more effective to ask about and use occupation as a control for material disadvantage in health studies.

## Endnote

^a^Data available from UK Data Service http://ukdataservice.ac.uk/.

## Competing interests

The authors declare that they have no competing interests.

## Authors’ contributions

CD identified the approach and developed the methodology, TC carried out the analysis and produced a first draft of the paper. TC and CD jointly revised the paper and are equally responsible for the intellectual insights. Both authors read and approve the final manuscript.

## Pre-publication history

The pre-publication history for this paper can be accessed here:

http://www.biomedcentral.com/1471-2288/14/59/prepub

## Supplementary Material

Additional file 1 Detailed description of modelling approach. Click here for file
