# Supplementary material for: A method for estimating wage, using standardised occupational classifications, for use in medical research in the place of self-reported income
Source: BMC Med Res Methodol. 2014 Apr 28;14:59. doi: 10.1186/1471-2288-14-59 (PMC4021181; doi:10.1186/1471-2288-14-59)
Supplement: Additional file 1 — Detailed description of modelling approach. [file 1471-2288-14-59-S1.docx]

**Additional file 1: Information on methodology**

We propose a multi-level mixed effects approach to model log transformed wages. This allows the calculation of Empirical Bayes estimates of wage for the SOC units. This estimator has the helpful property of accounting for sample size (ie the number of cases within the LFS for a particular SOC unit). Where the sample size is small the estimator will be pulled or ‘shrunk’ towards the mean of the higher level grouping within which it lies and ultimately the national average wage. In order to ensure that the resulting estimates can be replicated in as wide a range of data sources as possible, it was decided to restrict the variables used in the models to age, sex and occupation from the SOC. The mixed effects models utilise the tiered structure of the SOC and estimate random effect parameters associated with each of the groups within corresponding tiers of the SOC together with fixed effect parameters for age and sex. We compared a number of mixed models with random intercepts as well age varying slopes of the within SOC level regression lines and determine the best fitting of these models. For the purposes of this study, the levels within the multi-level model were defined as follows; level one refers to the individual (subscripted with i in the equations), level two to the 353 unit groups of SOC (subscripted with j) and level three to the 81 minor groups of SOC (subscripted with k). We calculated a geometric mean and variance of our wage measure, as a null model, to which subsequent models are compared.

The first model consisted of a 2-level mixed model with random intercepts and is shown in equation 1.

| log(wage_ij_) = β_1j_ + β_2_age_ij_ + β_3_sex_ij_ + Ɛ_ij_ | (1) |
| --- | --- |
| where β_1j =_ β_1 +_ ζ_1j_ |  |

Where wage_ij_ is the weekly wage for the i^th^ individual in the j^th^ SOC minor group and β_2_age_ij_ and β_3_sex_ij_ are the fixed age and sex coefficients respectively for the i^th^ individual in the j^th^ SOC minor group. The β_1j_ term contains the random effects portion of the model and is decomposed into j SOC minor group specific random intercepts ζ_j_ which deviate from β_1_ which represents the grand sample intercept. Ɛ_ij_ then represents the residual error term corresponding to the deviation of the i^th^ individual’s wage from β_1j_. This model can be understood as containing a ‘correction term’ in the random portion of the model which adjusts the predicted values for the variation in wages across SOC unit groups. The second model is identical to the first except for the additional decomposition of the random effect to incorporate an interaction between age and the level 2 SOC minor groups. The slopes, as well as the intercepts, for the SOC minor groups can therefore vary. The model thus allows for the differential effect of age across occupational groups at the SOC minor level. The equation for this model is given by:

| log(wage_ij_) = β_1j_ + β_2_age_ij_ + β_3_sex_ij_ + Ɛ_ij_ | (2) |
| --- | --- |
| where β_1j =_ β_1_ + ζ_1j_ + ζ_2j_age_ij_ |  |

In this model the error term Ɛ_ij_ comprises the deviation of the i^th^ individual’s wage from the j^th^ SOC minor specific regression line with slope ζ_2j_age_ij_ . The addition of ζ_2j_age_ij_ to the random effects part of the equation allows the strength and direction of an age effect to vary across SOC minor groups relative to the fixed portion of the model. The final two models include more detailed information from the SOC by including information from the unit classification level. Equation three includes random intercept terms (ζ_kj_ and ζ_k_) for both levels and equation four includes both random intercepts (ζ_1kj_ and ζ_1k_) and age slopes (ζ_2kj_age_ikj_ and ζ_2k_age_ikj_) for both levels.

| log(wage_ikj_) = β_1jk_ + β_2_age_ikj_ + β_3_sex_ikj_ + Ɛ_ikj_ | (3) |
| --- | --- |
| where β_1jk =_ β_1 +_ ζ_kj_ + ζ_k_ |  |
|  |  |
| log(wage_ikj_) = β_1kj_ + β_2_age_ikj_ + β_3_sex_ikj_ + Ɛ_ikj_ | (4) |
| where β_1kj_ = β_1_ + ζ_1kj_ + ζ_2kj_age_ikj_ + ζ_1k_ + ζ_2k_age_ikj_ |  |
